# Psychological distress, oral health behaviour and related factors among adolescents: Finnish School Health Promotion Study

**DOI:** 10.1186/s12903-020-01357-3

**Published:** 2021-01-06

**Authors:** Vesa Pohjola, Meri Nurkkala, Jorma I. Virtanen

**Affiliations:** 1 Faculty of Medicine, University of Oulu, Oulu, Finland; 2 Medical Research Center Oulu, Oulu University Hospital, Oulu, Finland; 3 Department of Clinical Dentistry, Faculty of Medicine, University of Bergen, Bergen, Norway; 4 Institute of Dentistry, University of Turku, Turku, Finland

**Keywords:** Adolescents, Oral health behaviour, Psychological distress, Tooth brushing

## Abstract

**Background:**

Psychological distress may affect health behaviour. We examined how psychological distress, social phobia (SP) and anxiety associated with tooth brushing among Finnish adolescents with respect to gender, school grade, parents’ education, family structure, smoking and perceived general health.

**Methods:**

This study is part of the Finnish national School Health Promotion Study (SHP). The study population comprised a representative sample of Finnish 15-year-olds (N = 45,877). Mini-Social Phobia Inventory (Mini-SPIN) and generalized anxiety disorder (GAD) served to assess SP and anxiety. A questionnaire enquired about the respondents’ oral health habits (tooth brushing, smoking), background factors (age, gender, family structure and parents’ education) and perceived general health. Chi-squared tests and logistic regression analyses served in the statistical analyses.

**Results:**

About two-thirds of the girls (66.7%) and less than half of the boys (40.1%) followed the international recommendation of tooth brushing twice daily. Girls reported possible problems with SP and GAD more often than boys did. Those reporting possible problems with SP or moderate or severe anxiety brushed their teeth at least twice daily less often than did those reporting no possible problems with SP and those with no, slight or mild anxiety. Logistic regression analyses showed that male gender (OR = 3.2; 95% CI 3.1–3.4), parents’ basic education (OR = 1.5; 95% CI 1.4–1.5), and adolescents’ perception of their current state of health as moderate, fairly or very poor (OR = 1.8; 95% CI 1.5–2.0) associated with not brushing teeth twice daily. Gender-specific logistic regression analyses showed that boys who smoked (OR = 1.7; 95% CI 1.6–1.8) were less likely than non-smokers to brush their teeth twice daily.

**Conclusion:**

Adolescents with psychological distress, such as possible SP or possible general anxiety, had less favourable oral health behaviour. Psychological distress indicates a greater risk for oral health problems already in adolescence.

## Background

Oral health is an important part of overall wellbeing, and oral diseases are related to chronic health conditions such as diabetes and cardiovascular diseases [1, 2]. The common risk factors of oral and general heath have been highly influential in integrating oral health into general health [3]. The common risk factor approach has been connected into a social determinants framework and the theories explaining oral health inequalities have been incorporated into one conceptual framework (CF) [3]. The CF describes how the social determinates affect oral health through structural and intermediary determinants, which include material, behavioural, cultural and psychosocial determinants [3, 4].

Many psychological, social and physical changes occur in adolescence. As the requirement for independent functioning increases in adolescence, anxiety and social phobia (SP) may affect young person’s ability to cope and can adversely impact one’s quality of life [5, 6]. Symptoms of anxiety and SP often emerge during late childhood or adolescence [7]. Girls tend to suffer from psychological distress more often than boys do [8, 9]. The Mini-Social Phobia Inventory (Mini-SPIN) can be used to identify SP among adolescents [7], and Generalized Anxiety Disorder (GAD), one of the most common psychiatric disorders [10], can be assessed with the GAD-7 questionnaire [11, 12].

The basis for oral health behaviours and practices is developed at a young age. The transition period from childhood to adulthood is characterised by many changes [13]. During this period, independence from parents increases, resulting in changes in behaviour, including those relating to eating habits, smoking, health behaviour and lifestyle behaviour [13, 14]. The development of these independent behaviours can affect oral health and establish life-long patterns of behaviour [13]. Young people who take care of their teeth tend to promote other dimensions of their health as well [15, 16].

Tooth brushing is the most effective oral hygiene method, and the universally recommended frequency is to brush one’s teeth twice daily [17]. Tooth brushing among adolescents reflects a gender difference, as girls brush their teeth more often than boys do [15, 16, 18–20]. High occupational status of the parents and family affluence have shown a connection to higher prevalence among young adolescents of tooth brushing more than once-a-day [19]. Studies have also shown smoking to associate negatively with socio-educational backgrounds and to connect strongly with infrequent tooth brushing habits among adolescents [18, 21]. Children living in two-parent families show a have higher prevalence of tooth brushing more than once-a-day [19].

Studies have shown relationships between psychological distress and dental health among adults, including the association of depression and anxiety with lower tooth brushing frequency [22], and the relationship that stress and depressive symptoms have with poor oral health [22–24]. Studies conducted outside of Europe have reported that adolescents with psychological distress brush their teeth twice daily less often than do adolescents with good mental health [25]. The association between periodontitis and dimensions of psychosocial distress has be documented already in adolescence [26] and poor maternal factors (e.g. low education, and psychological distress) can have a cumulative impact on future caries experience of adolescents [27].

The evidence of how psychological distress may affect oral health behaviour among adolescents is scarce. We aimed to study the association between tooth brushing and psychological distress among Finnish adolescents while controlling for gender, parental education, family structure, smoking and perceived general health. We hypothesised that those with more psychological distress would brush their teeth twice daily less often than would those with less psychological distress.

## Methods

### Subjects

This study uses data from the nationwide Finnish School Health Promotion study (SHP), which monitors the health, health behaviour, wellbeing and schooling of 14- to 20-year-olds in Finland. The SHP study is carried out nationwide every second year. Respondents include pupils in their eighth and ninth years of comprehensive school in mainland Finland and the Åland Islands, covering 80% of this target group in Finland [28]. Every municipality in Finland receives the survey and decides whether the schools in their area will participate in it [29]. The Ethics Committee of the National Institute for Health and Welfare, Finland, approved the study. Participation in the study was completely voluntary, and students consented to participate by answering the survey.

The data were collected in April 2013 with an anonymous, voluntary questionnaire administered in the classroom under a teacher’s supervision [28]. In Finland, comprehensive school lasts nine years: from age 7 to age 16 years.

In 2013, the SHP study covered participants from the whole country and the participation rate was 84% among the adolescents in eight and ninth grades of comprehensive school [15]. For the present cross-sectional study, we obtained data from 45,877 15-year-olds (excluding 830 participants who failed to report their tooth brushing frequency) who in the spring of 2013 were in their eighth (37%) or ninth (63%) year of comprehensive school. Males comprised 49.9% of our study sample.

### The questionnaire

The questionnaire enquired about factors related to the students’ health with several questions. The question ‘How often do you brush your teeth?’ enquired about tooth brushing frequency with the following answer options: ‘never’; ‘less than once a week’; ‘at least once a week but not daily’; ‘once daily’; or ‘more than once daily’. For our analyses, we formed three classes (‘less than once daily’, ‘once daily’, ‘twice or more daily’), and dichotomised (‘less than twice daily, ‘at least twice daily’) variables according to the international recommendation to brush one’s teeth twice daily [17]. In the CF tooth brushing was seen as a behavioural determinant.

The questionnaire enquired about the participants’ gender and year in school (8th or 9th year of comprehensive school).

The question ‘What is the highest educational level your parents have achieved?’ enquired about the parents’ highest education level (separately for the mother and father) with the following answer options: ‘primary or comprehensive school’; ‘upper secondary school or vocational education’; ‘occupational studies in addition to upper secondary school or vocational education’; ‘university, university of applied sciences, or other higher education institution’; or ‘no education’. We further placed these alternatives into three categories by combining the first and last options (‘basic education or less’), as well as the second and third options (‘upper secondary school or vocational education with or without occupational studies’). For the logistic regression analyses, we dichotomised categories into two (‘secondary or tertiary education’, ‘basic education or less’). The question ‘Who are the adults you live with?’ aimed to determine the participants’ family structure with the following answer options: ‘my mother and my father’; ‘my mother and my father alternately, my parents don’t live together’; ‘only my mother’; ‘only my father’; ‘my father/mother and his/her partner’; ‘one or more other adults; or ‘none of the above’. We dichotomised these alternatives into ‘with both parents (mother and father)’ or ‘other’, assuming that living with both parents would stand out from other family structures in terms of health behaviour [30]. In the CF parental education was considered as a material determinant and family structure as a material or social determinant.

The question ‘Which of the following alternatives best describes your current smoking habits?’ assessed the adolescents’ smoking habits among those who had ever smoked with the response alternatives: ‘I smoke once or more daily’; ‘I smoke once or more a week, but not every day’; ‘I smoke less than once a week’; or ‘I have quit smoking (temporarily or permanently)’. Respondents who answered the question ‘How many cigarettes, pipefuls of tobacco and cigars have you smoked altogether?’ with the answer option ‘none’ were identified as non-smokers. We then formed two categories for current smoking habits (‘daily or occasional smoker’, ‘non-smoker’). Smoking was seen as a behavioural determinant in the CF.

The question ‘How is your health in general?’ aimed to determine the respondents’ perceived overall health with the following four answer options: ‘very good’, ‘fairly good’, ‘moderate’, or ‘fairly or very bad’. We further dichotomised the alternatives for the regression analyses (‘very good, fairly good or moderate’; ‘fairly or very bad’). Since oral health and general health are interconnected in the CF, the question of general health was used as an indicator of health.

We assessed psychological distress with the Mini-Social Phobia Inventory (Mini-SPIN), a three-item self-rated scale used as a screening tool to help identify individuals at increased risk for SP [5]. The Mini-SPIN has proved valid in identifying possible SP among adolescents, and a score of 6 points or greater was found optimal in predicting SP with a sensitivity of 86% and specificity of 84% [7]. The Mini-SPIN includes the question ‘How much have the following problems bothered you during the past week?’ with three responses: (1) ‘Fear of embarrassment causes me to avoid doing things or speaking to people’, (2) ‘I avoid activities in which I am the centre of attention’ and (3) ‘Being embarrassed or looking stupid are among my worst fears’. The items are rated on a Likert scale: 0 = Not at all, 1 = A little bit, 2 = Somewhat, 3 = Very much and 4 = Extremely. The cut-off score for a possible SP diagnosis is six or more points [5, 7]. Based on this cut-off score, we formed two categories (0–5 points: no SP, 6–12 points: possible SP). The Mini-SPIN can serve as a primary assessment of one’s social anxiety; the results can help to identify any need for further examination.

The questionnaire also included the reliable and validated GAD-7 questionnaire [11, 12], which served in screening for Generalized Anxiety Disorder (GAD). GAD-7 has demonstrated good psychometric properties in adolescents in Finland, the internal consistency of GAD-7 has been good (Cronbach's α = 0.91); the associations of GAD-7 sum scores with self-report measures of depression and social anxiety have supported construct validity [12]. The questionnaire contains seven items, including the question ‘During the past two weeks, how often have the following problems bothered you?” with the following answer options: (1) ‘Feeling nervous, anxious or on edge’, (2) ‘Inability to stop or control worrying’, (3) ‘Worrying too much about different things’, (4) ‘Trouble relaxing’, (5) ‘Being so restless that it’s hard to sit still’, (6) ‘Becoming easily annoyed or irritable’ and (7) ‘Feeling afraid, as if something awful might happen’, each rated on the following scale: 0 = Not at all, 1 = Several days, 2 = More than half the days, and 3 = Nearly every day. When screening for anxiety disorders, a cut-off score of ten or higher is recommended for further evaluation [11, 12]. We formed two categories for the regression analyses based on this cut-off score (0–9 points: no, slight or mild anxiety; 10–21 points: moderate to severe anxiety). Psychological distress was considered as a psychosocial determinant in the CF.

### Statistical analysis

After checking the distribution of the data we used cross tabulation with Chi-squared test to analyse associations between background variables, social distress (measured with Mini-SPIN and GAD-7) and tooth brushing frequency. After checking correlations and multicollinearity, we conducted logistic regression analyses with tooth brushing as the dependent variable, and age, gender, family structure, parents’ educational level, adolescent’s smoking status, adolescent’s perceived general health and social distress (measured with Mini-SPIN) as covariates. Because gender strongly impacted tooth brushing, we conducted gender-specific logistic regression analyses as described above. We then repeated the logistic regression analyses using GAD-7 instead of Mini-SPIN as a measure of psychological distress. We presented the results with adjusted odds ratios (OR) and their 95% confidence intervals (95% CI). We used IBM SPSS Statistics 22 for all statistical analyses and considered p values < 0.05 statistically significant.

## Results

Table 1 shows tooth brushing among 15-year-old Finns according to background factors. About two thirds of the girls (66.7%) and less than half of the boys (40.1%) followed the international recommendation of twice daily tooth brushing. Adolescents whose parents had a basic education brushed their teeth twice daily less often than did adolescents whose parents had a secondary or tertiary education. Also, adolescents living with both parents brushed their teeth twice daily more often than did adolescents living in other family structures. Smokers brushed their teeth twice daily less often than non-smokers did.

**Table 1:** Tooth brushing frequency among Finnish 15-year-olds (%, n) by their characteristics

	Tooth brushing		
	≤ Once daily % (n)	≥ Twice daily % (n)	Total % (n)
*Gender*			
Boys	59.9 (13,714)	40.1 (9177)	100 (22,891)
Girls	33.2 (7645)	66.7 (15,341)	100 (22,986)
All	46.6 (21,359)	53.4 (24,518)	100 (45,877)
*School grade*			
8th grade	48.1 (8149)	51.9 (8808)	100 (16,957)
9th grade	45.7 (13,210)	54.3 (15,710)	100 (28,920)
*Parents’ education*			
Basic education or less	56.2 (1498)	43.8 (1166)	100 (2664)
Secondary education^a^	49.8 (10,244)	50.2 (10,310)	100 (20,554)
Tertiary education^b^	40.9 (7966)	59.1 (11,509)	100 (19,475)
*Family structure*			
With both parents	44.4 (13,565)	55.6 (17,018)	100 (30,583)
Other	50.8 (7322)	49.2 (7092)	100 (14,414)
*Current smoking habits*			
Daily or occasional	54.2 (6372)	45.8 (5387)	100 (11,759)
Non-smoker	43.9 (14,691)	56.1 (18,796)	100 (33,487)
*How is your health in general? Is it*			
Very good	41.9 (6471)	58.1 (8984)	100 (15,455)
Fairly good	46.8 (10,752)	53.2 (12,201)	100 (22,953)
Moderate	54.5 (3397)	45.5 (2838)	100 (6235)
Fairly or very bad	62.5 (610)	37.5 (366)	100 (976)

^a^Secondary education = high school, vocational school, occupational studies

^b^Tertiary education = University, polytechnics

(p < 0.001 for all associations; chi-square test)

**Fig. 1:**
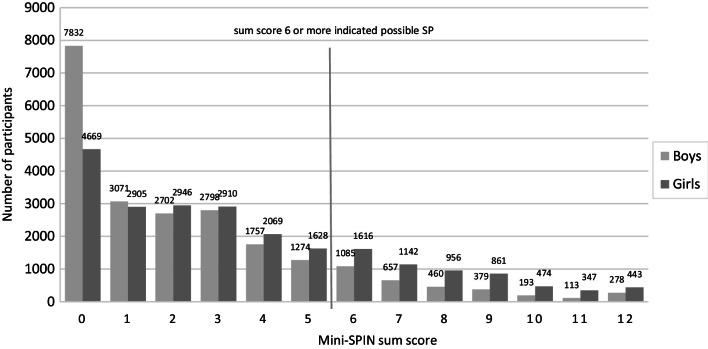
The number of participants according to the Mini-SPIN sum score

**Fig. 2:**
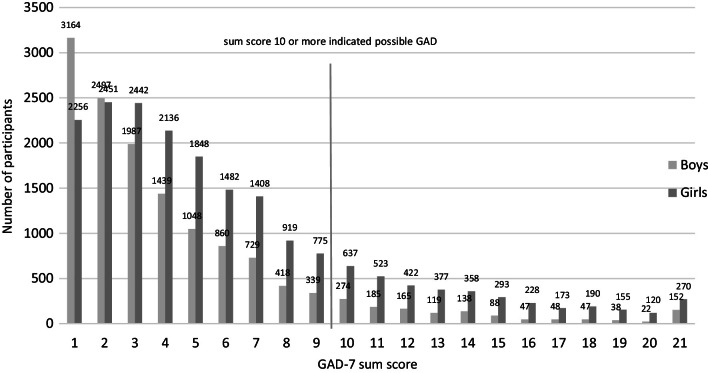
The number of participants according to GAD-7 sum score

The sum scores of the Mini-SPIN and GAD-7 questionnaires among the Finnish 15-year-olds are presented in Figs. 1 and 2. Girls reported possible problems with SP and GAD more often than boys did: among the girls 25.4% and among the boys 14.0% had Mini-SPIN sums core 6 or more indicating possible SP. Regarding GAD-7 16.6% of the girls and 6.0% of the boys had sum score 10 or more indicating possible GAD. The correlation between the three Mini-SPIN items and the seven GAD-7 items was up to 0.4. The most frequently reported Mini-SPIN item was ‘Being embarrassed or looking stupid are among my worst fears’, with nearly one in five girls (19.1%) and one in ten boys (10.1%) answering ‘Very much’ or ‘Extremely’ for this item (data not presented). The most frequently reported item in the GAD-7 questionnaire was ‘Becoming easily annoyed or irritable’, with nearly a quarter of the girls (24.9%) and one in ten (10.8%) boys answering ‘More than half of the days’ or ‘Nearly every day’ for this item (data not presented).

Those boys and girls (Table 2) reporting possible problems with SP brushed their teeth at least twice daily less often than did those reporting no possible problems with SP. Of those reporting problems with SP, seven in ten boys and four in ten girls brushed their teeth once daily or less. Also, those reporting moderate or severe anxiety (GAD-7 score ≥ 10) brushed their teeth at least twice daily less often than did those with no, slight or mild anxiety (GAD-7 score ≤ 9).

**Table 2:** Tooth brushing (%/n) among Finnish 15-year-old boys and girls according to Mini-SPIN and GAD-7 classification

	Tooth brushing		
	≤ Once daily % (n)	≥ Twice daily % (n)	Total % (n)
*Boys*			
Mini-SPIN classification			
0–5 points (No SP)	58.8 (11,175)	41.2 (7828)	100 (19,003)
6–12 points (Possible SP)	66.4 (2059)	33.6 (1043)	100 (3102)
Total	59.9 (13,234)	40.1 (8871)	100 (22,105)
GAD-7, 2 categories			
0–9 points (No, slight or mild anxiety)	59.2 (11,937)	40.8 (8230)	100 (20,167)
10–21 points (Moderate to severe anxiety)	68.2 (876)	31.8 (408)	100 (1284)
Total	59.7 (12,813)	40.3 (8638)	100 (21,451)
*Girls*			
Mini-SPIN classification			
0–5 points	31.1 (5267)	68.9 (11,677)	100 (16,944)
6–12 points	39.6 (2286)	60.4 (3492)	100 (5778)
Total	33.2 (7553)	66.8 (15,169)	100 (22,722)
GAD-7, 2 categories			
0–9 points	31.9 (5955)	68.1 (12,691)	100 (18,646)
10–21 points	39.6 (1462)	60.4 (2229)	100 (3691)
Total	33.2 (7417)	66.8 (14,920)	100 (22,337)

(p < 0.001 for all associations; chi-square test)

Logistic regression analyses (Table 3) showed that boys were more likely than girls (OR = 3.2; 95% CI 3.1–3.4) not to brush their teeth twice daily. Adolescents whose parents had only a basic education (OR = 1.5; CI 95% 1.4–1.5) were also more likely than adolescents whose parents had a secondary or tertiary education not to brush their teeth twice daily. Adolescents reporting a good or very good perceived current state of health brushed their teeth twice daily more often than did adolescents reporting a moderate, fairly or very bad perceived current state of health.

**Table 3:** Results of logistic regression analyses tooth brushing (n = 45,877) as dependent variable

	OR	95% CI
*Sex*		
Girl	1.0	
Boy	3.2*	(3.1–3.4)
*Mini-SPIN*		
0–5 points (No SP)	1.0	
6–12 points (Possible SP)	1.4*	(1.3–1.4)
*Parents’ education*		
Secondary or tertiary education	1.0	
Basic education or less	1.5*	(1.4–1.5)
*Family structure*		
With both parents	1.0	
Other	1.2*	(1.2–1.3)
*Smoking*		
Non-smoker	1.0	
Daily or occasional	1.5*	(1.4–1.5)
*How is your health in general? Is it*		
Very good, fairly good or moderate	1.0	
Fairly or very bad	1.8*	(1.5–2.0)

Once daily or less often = 1

Nagelkerke R Square 0.116

*p < 0.001

Gender-specific logistic regression analyses (Table 4) showed that those reporting possible problems with SP were less likely than those not reporting possible problems with SP to brush their teeth twice daily: boys (OR = 1.3; 95% CI 1.2–1.5) and girls (OR = 1.4; 95% CI 1.4–1.5). Especially among boys, smokers (OR = 1.7; 95% CI 1.6–1.8) were less likely than non-smokers to brush their teeth twice daily. Those reporting poor perceived general health were less likely than those reporting moderate or good perceived general health to brush their teeth twice daily: girls (OR = 1.9; 95% CI 1.6–2.2) and boys (OR = 1.6; 95% CI 1.2–2.1). The gender specific logistic regression analyses with GAD-7 showed similar findings (Table 5) to those with Mini-SPIN (described above).

**Table 4:** Gender specific results of logistic regression analyses including Mini-SPIN tooth brushing (n = 45,877) as dependent variable

	Boys		Girls	
	OR	95% CI	OR	95% CI
*Mini-SPIN*				
0–5 points (No SP)	1.0		1.0	
6–12 points (Possible SP)	1.3*	(1.2–1.5)	1.4*	(1.3–1.5)
*Parents’ education*				
Secondary or tertiary education	1.0		1.0	
Basic education or less	1.5*	(1.4–1.6)	1.5*	(1.4–1.6)
*Family structure*				
With both parents	1.0		1.0	
Other	1.2*	(1.2–1.3)	1.2*	(1.2–1.3)
*Smoking*				
Non-smoker	1.0		1.0	
Daily or occasional	1.7*	(1.6–1.8)	1.3*	(1.2–1.3)
*How is your health in general? Is it*				
Very good, fairly good or moderate	1.0		1.0	
Fairly or very bad	1.6*	(1.2–2.1)	1.9*	(1.6–2.2)

Once daily or less often = 1

Boys: Nagelkerke R Square 0.031

Girls: Nagelkerke R Square 0.023

*p < 0.001

**Table 5:** Gender specific results of logistic regression analyses including GAD-7 tooth brushing (n = 45,877) as dependent variable

	Boys		Girls	
	OR	95% CI	OR	95% CI
*GAD-7*				
0–9 points (No, slight or mild anxiety)	1.0		1.0	
10–21 points (Moderate to severe anxiety)	1.2*	(1.1–1.4)	1.2*	(1.1–1.3)
*Parents’ education*				
Secondary or tertiary education	1.0		1.0	
Basic education or less	1.5*	(1.4–1.6)	1.5*	(1.4–1.6)
*Family structure*				
With both parents	1.0		1.0	
Other	1.3*	(1.2–1.3)	1.2*	(1.2–1.3)
*Smoking*				
Non-smoker	1.0		1.0	
Daily or occasional	1.7*	(1.6–1.8)	1.2*	(1.1–1.3)
*How is your health in general? Is it*				
Very good, fairly good or moderate	1.0		1.0	
Fairly or very bad	1.7*	(1.3–2.3)	1.9*	(1.5–2.2)

Once daily or less often = 1

Boys: Nagelkerke R Square 0.029

Girls: Nagelkerke R Square 0.018

*p < 0.001

## Discussion

Psychological distress associated with tooth brushing among Finnish adolescents. Those reporting possible problems with SP or moderate to severe possible general anxiety brushed their teeth twice daily less often than do those reporting no possible problems with SP or reporting no or low general anxiety. Although the association between tooth brushing and psychological distress was not strong, the difference between those having or not having possible psychological distress and brushing their teeth twice daily varied between 7.6 and 9%, which is clinically important. Our results support the results of previous studies of adolescents in Asia, Africa, Caribbean and South America [25]. The problems causing psychological distress may dominate so much of an adolescent’s attention that they become unable to follow the recommended oral health behaviour. The main factors that motivate tooth brushing (along with caries prevention) are social benefits: clean teeth look attractive, create a feeling of freshness and strengthen one’s self-confidence [31]. Fewer social problems could make adolescents more aware of the social benefits of tooth brushing and thus increase their motivation to regularly brush their teeth. Possible problems with SP indicate a higher risk for oral health problems among adolescents.

Twice daily tooth brushing prevalence was low especially among the Finnish boys. However, some improvement in tooth brushing has occurred: while 45% of the Finnish 15-year-olds brushed according to the recommendation in 2001–2002, the corresponding figure in our study was 53% [32]. As in previous studies [8, 9], girls in this study reported possible SP and anxiety more often than boys did. More frequent problems with SP among girls could lead to negative oral health effects due to psychological distress. However, girls also reported brushing their teeth twice daily more often than boys did, offering girls greater protection from oral health problems. In addition to psychological distress, other factors may affect girls’ frequency of tooth brushing. Girls may, for example, consider clean, attractive-looking teeth and oral health more important than boys do, which could improve their mood and increase their motivation for tooth brushing. Additionally, women generally engage in positive health behaviours more often than men do [33].

In this study, adolescents whose parents had only a basic education brushed their teeth twice daily less often than did adolescents whose parents had a secondary or tertiary education. Earlier studies have also found that a parent’s higher educational level and socioeconomic status associate with an adolescent’s more frequent tooth brushing [19, 34]. Higher parental educational level has positively associated with children’s psychological health and reduced health complaints [35], which could also stem from a good socioeconomic situation. Additionally, higher educational expectations of the family have shown a link to better health behaviour, including tooth brushing [36].

The results of this study suggest that family structure associates with tooth brushing; those living in a traditional family of two parents brushed their teeth twice daily more often than did those living in a different family setting. Earlier studies of the relationship between family structure and children’s tooth brushing habits have shown varying results in different countries [19]. Parents strongly affect their children’s tooth brushing habits; regular family routines and practices promoting good oral health early in life are important for promoting children’s oral health [19, 37]. Regular family routines can be more easily achieved in a family of two parents than in, for example, a family of one parent. In addition, socioeconomically disadvantaged adolescents often suffer from social stressors such as dysfunctional family relationships and household dynamics, which could increase their risk for poor oral health [37]. People with mental health disorders may also have limited capacity for self-care and preventing oral diseases. In addition, providing oral health care for people with mental health disorders is challenging [38].

In this study, smoking associated with poor tooth brushing, especially among boys. Earlier studies have shown similar findings among boys and girls [18, 39] and among young men [40]. In general, smokers tend to have poorer oral health habits and oral health than non-smokers have [40]. Because smoking affects one’s sense of smell and taste, smokers may not feel the clean and fresh feeling that tooth brushing gives in the same positive way as non-smokers do; they may also consider oral heath less important than non-smokers do.

The results of this study showed that adolescents reporting good or very good perceived health brushed their teeth twice daily more often than did adolescents reporting moderate, fairly or very poor perceived health. Since tooth brushing is connected to oral health, which associates with general health, that those reporting compromised perceived health brushed their teeth less than twice daily was unsurprising. Few studies have examined the association between tooth brushing and general heath. One recent study of 71 449 participants found that a higher frequency of daily tooth brushing reduced the development of all types of malignancies [41]. Additionally, because poor health behaviours tend to cluster together [16, 18, 39], infrequent tooth brushing, together with other poor health habits and other aspects of life, may contribute to poor perceived health.

The Finnish School Health Promotion Study has a large sample size covering approximately 80% of Finnish 15-year-olds with an equal gender distribution, and its findings may be generalised to all Finnish 15-year-olds. The large sample size also makes our study comparable to international surveys of adolescents’ oral health behaviour. Because the Finnish School Health Promotion Study takes place regularly (every 2 years), it provides important information about the development of adolescents’ health behaviour. The use of validated instruments, such as the Mini-SPIN and the GAD-7, to assess the participants’ SP and anxiety increased the study’s reliability and validity. Self-reported outcome measures may be susceptible to socially desirable answering, but because participants answered voluntarily and anonymously, the results can be considered reliable. Because this study is cross-sectional, one should make no causal interpretations.

## Conclusion

In conclusion, adolescents reporting possible problems with SP or possible general anxiety brushed their teeth twice daily less often than did those reporting no possible problems with SP or reporting no general anxiety. Psychological distress indicates a higher risk for oral health problems already in adolescence and may in part explain oral health inequalities later in life.

## Data Availability

Data is available from the National Institute for Health and Welfare (THL), Finland where registered researchers may apply data according to the conditions set for this data. https://thl.fi/en/web/thlfi-en/research-and-expertwork/population-studies/school-health-promotion-study.

## Abbreviations

GAD: Generalized anxiety disorder
GAD-7: Generalized Anxiety Disorder Questionnaire
Mini-SPIN: Mini-Social Phobia Inventory
SHP: School Health Promotion Study
SP: Social phobia
